# ADAM9 is highly expressed in renal cell cancer and is associated with tumour progression

**DOI:** 10.1186/1471-2407-8-179

**Published:** 2008-06-26

**Authors:** Florian R Fritzsche, Kirsten Wassermann, Monika Jung, Angelika Tölle, Ilka Kristiansen, Michael Lein, Manfred Johannsen, Manfred Dietel, Klaus Jung, Glen Kristiansen

**Affiliations:** 1 Institute of Pathology, Charité – Universitätsmedizin Berlin, Berlin, Germany; 2 Institute of Urology, Charité – Universitätsmedizin Berlin, Berlin, Germany; 3 Institute of Surgical Pathology, UniversitätsSpital Zürich, Zurich, Switzerland

## Abstract

**Background:**

**A D**isintegrin **A**nd **M**etalloprotease (ADAM) 9 has been implicated in tumour progression of various solid tumours, however, little is known about its role in renal cell carcinoma. We evaluated the expression of ADAM9 on protein and transcript level in a clinico-pathologically characterized renal cell cancer cohort.

**Methods:**

108 renal cancer cases were immunostained for ADAM9 on a tissue-micro-array. For 30 additional cases, ADAM9 mRNA of microdissected tumour and normal tissue was analyzed via quantitative RT-PCR. SPSS 14.0 was used to apply crosstables (Fisher's exact test and χ^2^-test), correlations and univariate as well as multivariate survival analyses.

**Results:**

ADAM9 was significantly up-regulated in renal cancer in comparison to the adjacent normal tissue on mRNA level. On protein level, ADAM9 was significantly associated with higher tumour grade, positive nodal status and distant metastasis. Furthermore, ADAM9 protein expression was significantly associated with shortened patient survival in the univariate analysis.

**Conclusion:**

ADAM9 is strongly expressed in a large proportion of renal cell cancers, concordant with findings in other tumour entities. Additionally, ADAM9 expression is significantly associated with markers of unfavourable prognosis. Whether the demonstrated prognostic value of ADAM9 is independent from other tumour parameters will have to be verified in larger study cohorts.

## Background

Renal cell cancer (RCC) is thought to cause 12.890 deaths in 2007 in the USA [1] and accounts for around 2–3% of cancers worldwide [2,3]. It is one of the most lethal urologic malignancies. Nodal and systemic metastasis as well as vascular invasion are important prognostic factors in this tumour entity [4]. New molecular markers are warranted to improve the classification of RCC, to provide further prognostic and predictive information and eventually to allow for an individualized cancer therapy [5-8].

In this study, we focused on ADAM9 (synonyms: MDC9, meltrin-γ), a member of the "**A D**isintegrin **A**nd **M**etalloprotease" family. Functionally, ADAMs participate in spermatogenesis, cell adhesion, myo- and neurogenesis, inflammation, cell migration and tissue remodelling [9,10]. ADAMs are membrane-anchored cell surface glycoproteins with a protease domain in addition to an adhesion domain. The structure of ADAMs was found to be related to soluble snake venom proteins which induce hemorrhage and basement membrane destruction [11,12]. The interactions of ADAMs with cell surface and extracellular matrix proteins like integrins and syndecans could be of relevance in tumour biology as these processes are vital for tumour progression defined by growth, invasion and metastasis [13-17]. Several ADAMs have been analyzed in various tumour entities and were often found to be differentially expressed, partially conveying prognostic information [18-32]. Several ADAMs have already been shown up-regulated in renal cancer on transcript level, with ADAM8 being associated with shortened survival times and distant metastasis [33,34].

ADAM9 has been proposed to be involved in the ectodomain shedding of membrane-anchored of heparin-binding epidermal growth factor-like growth factor, probably regulated by the binding protein Eve-1 [35-37]. Possible mediating effects on EGFR activity further support the notion of ADAM9 involvement in carcinogenesis and tumour progression [38,35,41]. Moreover, ADAM9 promotes cancer cell invasion by modifying or regulating e-cadherin and several types of integrins [21,42].

We evaluated the ADAM9 expression on protein and transcript level to clarify a diagnostic or prognostic value of ADAM9 in renal cell cancer. We found ADAM9 mRNA up-regulated in RCC and demonstrated a prognostic value of ADAM9 protein expression for overall survival times.

## Methods

### Patients (RT-PCR)

Thirty matched malignant and non-malignant kidney tissue samples were derived from patients (26 male, four female; mean age 62 years, range: 40 to 92 years) with clear cell (cc) RCC undergoing radical nephrectomy at the Department of Urology, Charité – Universitätsmedizin Berlin, between September 2003 and January 2006.

Cases used for mRNA isolation were different from the cohort used for immunohistochemistry. Thirteen of the 30 ccRCC were pT1 stage, two tumours were pT2, and 15 tumours were pT3. Histological grading: G1 (n = 3), G2 (n = 25) and G3 (n = 2). None of the patients had known nodal or distant metastasis according to preoperative screening (computed tomography of chest, abdomen and pelvis). Samples were collected immediately after surgery in tubes with RNAlater^® ^Stabilization Reagent (Qiagen, Hilden, Germany). Until RNA isolation the tubes were stored at 4°C overnight and then at -80°C until analysis.

### Patients (immunohistochemistry)

One-hundred-eight patients (83 men, 25 women) diagnosed for renal cancer at the Institute of Pathology, Charité – Universitätsmedizin Berlin between 2003 and 2005 were enclosed in this study. The study has been approved by the Charité University Ethics Committee under the title 'Retrospektive Untersuchung von Gewebeproben mittels immunhistochemischer Färbung und molekularbiologischer Methoden' ('Retrospective analysis of tissue samples by immunohistochemistry and molecular biological techniques') (EA1/06/2004) on 20 September 2004.

Patient age ranged between 28 and 92 years with a median of 62. Histological diagnosis was established according to the guidelines of the World Health Organization. Cases were selected according to tissue availability and were not stratified for any known preoperative or pathological prognostic factors. Eightysix (79.6%) patients had a clear cell RCC (ccRCC), 17 (15.7%) a papillary RCC and five (4.6%) a chromophobe RCC. Twentythree patients had systemic disease (pM1) at the time of diagnosis. Tumour specific survival times, as annually assessed, was available for all patients. The median follow-up time of all cases was 30 months, ranging from one to 47 months. Twentythree patients died from renal cancer. The pT stages: 54 (50.0%) pT1, 3 (2.8%) pT2, 47 (43.5%) pT3 and 4 (3.7%) pT4. Twelve patients (11.1%) had pathologically confirmed nodal metastases (pN1 = 3, pN2 = 9). Fiftyone (47.2%) patients had no nodal metastases (pN0). Of 45 (41.7%) patients no lymph nodes were histologically examined (pNx). Hemangiosis carcinomatosa (V1/2) was detected in 29 (26.8%) cases. Tumour grades: 11 (10.2%) G1, 75 (69.4%) G2, 18 (16.7%) G3 and 4 (3.7%) G4. The sample cohort for the immunohistochemical study was different from the sample cohort used for mRNA analysis. The grouped clinico-pathological data of the immunohistochemically analysed cases are described in the left columns of Table 1.

**Table 1:** Associations (χ^2^-tests/Fischers exact test) between the protein expression of ADAM9 in renal cell cancer and clinico-pathological parameters (percentages in brackets)

	**Total**	**low ADAM9**	**high ADAM9**	**p-value**
**All cases**	108 (100)	71 (65.7)	37 (34.3)	
**Age**				0.225
≤ 62	59 (54.6)	42 (71.2)	17 (28.8)	
> 62	49 (45.4)	29 (59.2)	20 (40.8)	
**Histology**				< 0.001
Clear cell	86 (79.6)	65 (75.6)	21 (24.4)	
chromophobe	5 (4.6)	2 (40.0)	3 (60.0)	
Papillary	17 (15.7)	4 (23.4)	13 (76.5)	
**pT-status**				0.104
pT1	54 (50.0)	40 (74.1)	14 (25.9)	
pT2/3/4	54 (50.0)	31 (57.4)	23 (42.6)	
pN-status*				0.022
pN0	51 (47.2)	36 (70.6)	15 (29.4)	
pN1	12 (11.1)	4 (33.3)	8 (66.7)	
**Grading**				0.002
G 1	11 (10.2)	11(100)	0 (0)	
G 2	75 (69.4)	50 (66.7)	25 (33.3)	
G 3/4	22 (20.4)	10 (45.5)	12 (54.5)	
**Residual tumour status**^#^				0.073
R0	78 (72.2)	56 (71.8)	22 (28.2)	
R1/2	15 (13.9)	7 (46.7)	8 (53.3)	
**Metastasis**				0.014
M0	85 (78.7)	61 (71.8)	24 (28.2)	
M1	23 (21.3)	10 (43.5)	13 (56.5)	

* 45 cases were pNx

^# ^15 cases were Rx

### Tissue micro array construction

A tissue micro array (TMA) was constructed as previously described [43]. Briefly, suitable areas for tissue retrieval were marked on standard haematoxylin/eosin (H&E) sections, punched out of the paraffin block and inserted into a recipient block. The tissue arrayer was purchased from Beecher Instruments (Woodland, USA). The punch diameter was 0.6 mm. The RCC array was constructed to represent 109 cases with two spots from the tumour and two spots representing matching normal tissue from the cortex region of the kidney. In four cases, the normal spots did not represent kidney tissue, leaving 105 cases with matched tumour and normal tissue, plus four cases with tumour only. The whole TMA was accomplished on three paraffin blocks.

### RNA Isolation

Total RNA was isolated from 50 mg RNAlater™ stabilised kidney tissue samples using the RNeasy Mini Kit (Qiagen) according to the manufacturer's instructions. Additionally, we introduced a DNase I (Qiagen) digestion step on the silica gel membrane of the spin column where the RNA was bound, washed and eluated. RNA was extracted with 30 μl RNase-free water and the RNA content was measured with the NanoDrop ND-1000 Spectrophotometer (NanoDrop Technologies, Wilmington, USA). The RNA integrity was validated with the RNA 6000 Nano LabChip^® ^kit on the Agilent 2100 Bioanalyzer (Agilent Technologies, Palo Alto, CA, USA). The Agilent 2100 Expert software generates so-called RNA Integrity Number (RIN) which is an accepted quality criterion for isolated RNA [44]. The RNA samples were stored at -80°C up to cDNA synthesis.

### First Strand cDNA Synthesis

cDNA synthesis was performed with the Transcriptor First Strand cDNA Synthesis Kit (Roche Applied Science, Penzberg, Germany) using 1 μg RNA in reaction. Kit-included random hexamer primers were applied for first strand cDNA synthesis after following procedure: 10 min at 25°C for primer annealing, 30 min at 55°C for reverse transcription step, 5 min at 85°C for inactivation of Transcriptor Reverse Transcriptase, then cooling on ice. The cDNA volume amounted to 20 μl. Tubes were stored at -20°C up to subsequent PCR. All cDNA samples were 1:5 diluted with RNase-free water for use as template in real-time PCR.

### Real-Time PCR

Real-time PCR was performed with the LightCycler Instrument (Roche). For relative mRNA quantification of target gene (ADAM9) two stably expressed reference genes, TBP (TATA box binding protein) and PPIA (Peptidyl isomerase A) were additionally determined [45]. The PCR reaction volumes for both reference genes were 10 μl and 20 μl for the target gene. All PCR reaction mixes included 1 μl diluted cDNA. The PCR run conditions for the TBP mRNA quantification were the same as described before [46]. For the reference gene PPIA and the target gene ADAM9 the PCR methods were also described previously [45]. The used primer/probe sequences (in 5'-3' direction) for ADAM9 mRNA quantification were as follows: forward primer: ggtgacagatttggcaattgtg, reverse primer: ttgtgccttcgttaaccatcc, donor probe: acgcctagtcgaggcaccaaatgttg-6Fl and the acceptor probe: Cy5.5-gtgtggatttccagctaggatcagatgttcc-P. The cDNA amplification was performed with the ready-to-use LightCycler^® ^FastStart DNA MasterPLUS HybProbe (Roche). Final reaction concentrations of both primers were 0.5 μmol/l and the concentration for the donor/acceptor probes were 0.2 μmol/l each. The PCR setup was: activation of FastStart Taq DNA Polymerase at 95°C for 15 min, followed by 45 cycles of denaturation at 95°C for 10 s, annealing at 62°C for 30 s and elongation at 72°C for 30 s. The temperature transition rate was in each cycle 20°C/s. The fluorescence detection was gained after each annealing step and data was evaluated by the method of second derivative maximum with the LightCycler Software 3.5 (Roche). To reduce the inter-run variability, the paired samples of non-malignant and malignant tissue areas were measured in one PCR run. Calibration curves for all three genes were generated with pooled cDNA. PCR efficiencies were calculated from cDNA dilution curves of pooled cDNA and amounted to 1.84 for PPIA, to 1.88 for TBP and to 1.95 for ADAM9. Each PCR run included a cDNA with known expression level and was used as standard for the quantification of the unknown samples calculated by LightCycler Software Version 3.5. Another pooled cDNA was used as run-to-run precision control. For relative quantification of ADAM9 mRNA the expressions were related to geometric mean of the two reference genes PPIA and TBP [47].

### Immunohistochemistry

Formalin fixed paraffin embedded tissue was freshly cut (3 μm). The sections were mounted on Superfrost slides (Menzel Gläser, Braunschweig, Germany), dewaxed with xylene and gradually hydrated. Antigen retrieval was achieved by pressure cooking in 0.01 M citrate buffer for 5 min. The primary ADAM9-antibody (goat polyclonal, AF949, R&D Systems, Wiesbaden, Germany) [20] was diluted 1:50 using a background reducing dilution buffer (Dako, Glostrup, Denmark) and incubated at room temperature for 1 hour. Detection took place with the REAL™ EnVision™ System (Dako) according to the manufacturer's instructions. Diaminobenzidin (Sigma-Aldrich, Munich, Germany) served as chromogen. Afterwards the slides were briefly counterstained with haematoxylin and mounted. As negative controls a set of sequential TMA slides was processed omitting the primary antibody to exclude unspecific background. To evaluate intratumoural heterogeneity of ADAM9 expression we additionally stained 10 conventional tissue slides with RCC.

### Evaluation of the immunohistochemical stainings

The immunostainings were evaluated by two genitourinary pathologists blinded for patient outcome using a multiheaded microscope. The staining intensity was evaluated with a four-tier grading system (0 = negative, 1 = weak, 2 = moderate and 3 = strong staining intensity). We used a 10% threshold to determine positivity. To delineate between low and high levels of ADAM9 expression the tumours with strong ADAM9 expression were grouped against those with none to moderate staining intensity. Additionally, we compared the difference of the ADAM9 immunoreactivity in the two tumour spots from each case.

### Statistical analysis

Statistical analysis was performed using SPSS, version 14.0. Fisher's exact tests, χ^2^-tests and Kruskal-Wallis tests were applied to assess the statistical significance of the associations between ADAM9 expression and clinico-pathological parameters. Rank correlations were calculated according to Spearman. Univariate survival analysis was carried out according to Kaplan-Meier, differences in survival curves were assessed with the Log rank test. Cox regression model was used for multivariate survival analyses. P values < 0.05 were considered significant.

## Results

### RNA quality and quantitative RT-PCR

**Figure 1:**
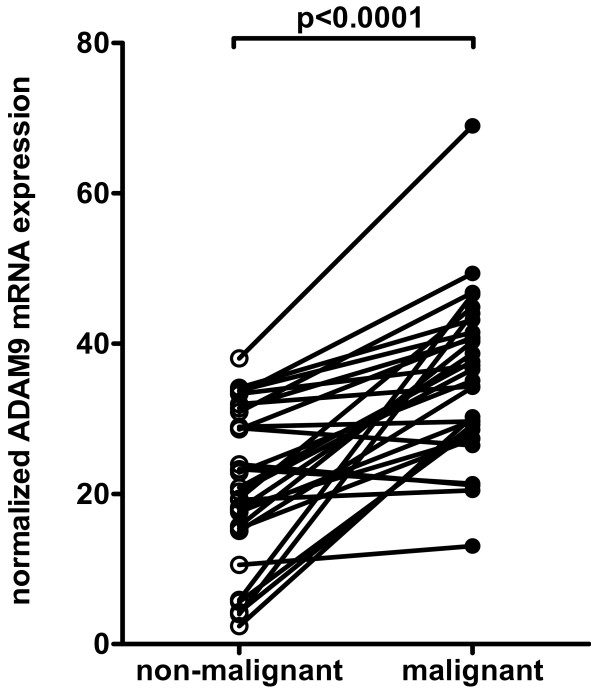
**ADAM9 mRNA expression**. ADAM9 mRNA expression of matched pairs of non-malignant and malignant renal tissue samples was normalized to the geometric mean of the two reference genes PPIA and TBP. For most cases mRNA levels of the renal cell cancers laid above those of the normal tissue standard.

RIN values of isolated RNA from kidney tissue samples ranged from 7.0 to 10.0 (mean = 8.7, SD = 0.80) reflecting the high quality and integrity of the RNA. The normalized ADAM9 expression was significantly higher in cancer than in normal tissue samples (mean change fold 2.7, range: 0.9–12.6; p < 0.0001; Figure 1). In three out of the 30 matched pairs ADAM9 mRNA expression was higher in the normal renal tissue. Significant associations between the mRNA expression of ADAM9 in RCC and clinico-pathological parameters (tumour stage, grading, nodal status, metastasis, histologic type and residual tumour status) could not be demonstrated (all p > 0.05).

### ADAM9 immunostaining in normal and malignant renal tissues

**Figure 2:**
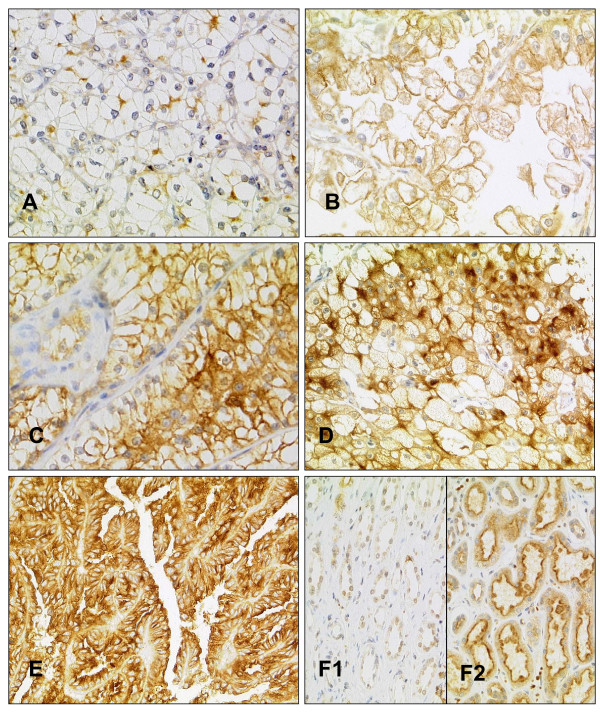
**ADAM9 immunohistochemistry**. **A-C **Clear cell carcinomas with weak (**A**), moderate (**B**) and strong (**C**) ADAM9 protein expression. **D/E **Papillary (**D**) and chromophobe (**E**) renal cell carcinoma with strong ADAM9 expression. **F **Normal renal tissue with weak (**F1**) and strong (**F2**) ADAM9 expression.

ADAM9 was expressed in malignant and non-malignant renal epithelia (Figure 2). The generally cytoplasmic immunoreactivity of ADAM9 was accentuated in the luminal part of the non-cancerous tubular epithelia and was particularly pronounced in the proximal tubules in comparison to distal tubules, which showed a weaker and more diffuse staining pattern. Glomerula and stromal cells were negative.

In renal cell carcinomas, a cytoplasmic and membranous staining was seen in all tumour subtypes, with papillary carcinomas displaying the strongest cytoplasmic immunoreactivity. The distribution of the ADAM9 staining is summarized in Table 2. One case swam off the slide during immunostaining. Although strong protein expression was found much more frequently in the tumour than in the normal tissue, these differences remained insignificant according to the Wilcoxon signed rank test (p = 0.367). The median of both tissue types was 2 and the mean 1.95 (normal) and 2.04 (tumour) respectively. Matched normal tissue was available in 104 cases. It is interesting to note that none of the normal tissues and only two of the cancer cases were completely negative for ADAM9.

**Table 2:** Distribution of ADAM9 staining in normal and malignant renal tissue (percentages in brackets)

**Staining**	**ADAM9 Normal**	**ADAM9 Cancer**
**Negative**	0 (0)	2 (1.9)
**1+**	9 (8.3)	29 (26.9)
**2+**	91 (84.3)	40 (37.0)
**3+**	4 (3.7)	37 (34.3)

Since the TMA was constructed to represent two spots of tumour from each case sampled from different tumour areas, differences of immunoreactivity between these spots can be used to estimate intratumoural heterogeneity of expression. We found ADAM9 staining completely equal both tumour spots in 91.7% (98/107; in one case only one spot had sufficient tumour tissue), which we consider a high rate of concordance. The additionally immunostained conventional tissue slides supported this estimation of a homogenous ADAM9 expression in tumour tissue. This is an important observation for it allows the use of TMAs for evaluation of ADAM9 in renal cell cancer.

### ADAM9 expression, clinico-pathological correlations and disease free survival times

In bivariate Spearman's rank correlations the ADAM9 protein expression in renal cancer correlated with the tumour grade, patient age and positive tumour resection status (R1) (Table 3). Additionally performed Kruskal-Wallis tests further confirmed the non-significant associations of ADAM9 expression with M-status (p = 0.096), nodal status (p = 0.098), whereas residual tumour status was of borderline significance (p = 0.050). In the χ^2^-tests higher ADAM9 protein expression was significantly associated tumour grade, distant metastasis, positive nodal status and papillary as well as chromophobe histologic subtype (Table 1).

**Table 3:** Correlation of ADAM9 protein expression in renal cell cancer with clinical/tumour-parameters

**ADAM9**	**ADAM9 N**	**pT-status**	**Grading**	**pN-status**	**R-status**	**M-status**	**Age**
CC	0.107	0.166	0.239	0.212	0.206	0.212	0.219
P	0.282	0.085	0.013	0.096	0.047	0.076	0.023
N	104	108	108	63	94	71	108

CC = Spearman's rank correlation coefficient, p = two sided significance, N = number of cases, ADAM9 N = ADAM9 expression in normal tissue, R-status = residual tumour status (R0/R1+), M-status = Metastasis (M0/M1).

**Figure 3:**
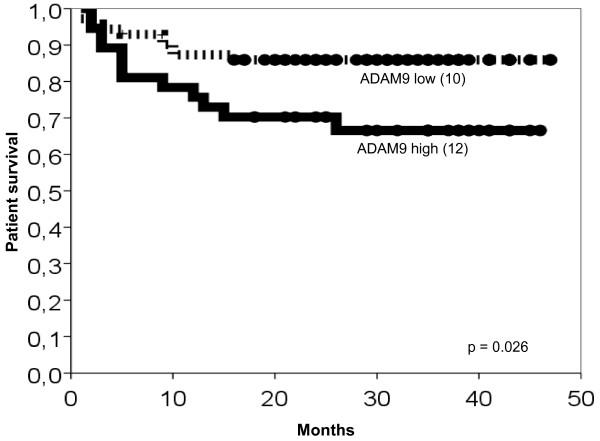
**Kaplan-Meier survival curves for ADAM9**. The number of events (deaths) is given in brackets. Tumours with high ADAM9 expression (bold line) revealed significantly shortened patient survival times if compared to those with low ADAM9 expression (dotted line).

In univariate analyses (Kaplan-Meier) of patient survival the clinico-pathological characteristics pT-status, grading, nodal status, residual tumour status (R0 vs. R1) and distant metastasis (M0 vs. M1) reached statistical significance (Table 4). Histologic subtype and age were no prognosticators of patient survival (data not shown). Higher ADAM9 expression in renal cancer was significantly associated with shortened survival times (Table 4 & Fig. 3, p = 0.026). The Cox multivariate survival analysis revealed no independent prognostic value for ADAM9 expression and tumour grading whereas pT-and pM-stage remained highly significant (Table 5). The univariate prognosticators pN- and residual tumour status were not included in the Cox regression model since because of missing data this would have led to a significant drop in the number of cases for this analysis.

**Table 4:** Univariate survival analysis (Kaplan-Meier)

**Characteristic**	**No. of cases**	**No. of events**	**Two-year survival rate (± SE) in %**	**p-value**
**ADAM9 expression**				0.026

low	71	10	85.9 ± 4.1	
high	37	12	70.3 ± 7.5	

**pT-status**				< 0.001

pT1	54	2	96.3 ± 2.6	
pT2/3/4	54	20	64.8 ± 6.5	

**Grading**				0.001

G 1	11	0	-	
G 2	75	12	85.3 ± 4.1	
G 3/4	22	10	54.5 ± 10.6	

**pN-status**				0.001

pN0	51	10	80.4 ± 5.6	
pN1+	12	8	41.7 ± 14.2	

**Residual tumour**				< 0.001

R0	78	10	87.2 ± 3.8	
R1/2	15	8	46.7 ± 12.9	

**Metastasis**				< 0.001

M0	85	8	90.6 ± 3.2	
M1	23	14	43.5 ± 10.3	

Survival times of patients with renal cancer according to clinico-pathological characteristics and ADAM9 protein expression.

**Table 5:** Multivariate survival analysis

**Variable**	**Relative Risk**	**95% CI**	**p-value**
ADAM9	1.081	0.433–2.696	0.868
pT-status	6.820	1.462–31.827	0.015
Grading	1.060	0.434–2.589	0.899
Metastasis	1.496	1.534–13.177	0.006

Cox regression model, n = 108: ADAM9 and clinico-pathological characteristics grouped as in Table 4. CI = confidence interval.

## Discussion

Several ADAMs, including ADAM9, have been described in various solid tumours on mRNA and/or protein level in and have often been associated with adverse prognostic parameters or shorter patient survival [18-21,23,25-32,48-50]. Our results are in line with this notion and demonstrate a prognostic value of ADAM9 for renal cell cancer at least in the univariate analysis. Although the whole model of the Cox analysis was significant, ADAM9 itself remained insignificant under multivariate conditions. Probably this might have been due to the strong association of ADAM9 expression with positive pM- status and higher tumour grade. Surprisingly, the histological tumour grade remained insignificant as well. This suggests that the cohort might have been not large enough to evaluate significance for these parameters, although pT-status and metastasis status could be verified as independent prognosticators. If nodal status and residual tumour status were included in the Cox analysis, all parameters would have lost their significance, probably caused by the strongly decreased number of cases in that analysis (data not shown).

ADAM9 was mainly expressed in the proximal part of the nephron. This is in line with a high expression of ADAM9 in clear cell and papillary carcinomas, which are thought to originate from that region. Interestingly also three of the five chromophobe RCC included showed a high ADAM9 expression, although this tumour type is thought to rather originate from the distal nephron. In our study we could not demonstrate a statistically significant up-regulation of ADAM9 protein expression in RCC in comparison to normal renal tissue. This is not in line with our results from the separate mRNA-sample cohort, where only 10% of the normal tissue displayed higher ADAM9 expression than the tumour. Possibly, posttranscriptional processes could be responsible for this discrepancy. On the other hand, only 4% of normal tissues showed a strong ADAM9 protein expression in comparison to 34% of the tumours, which clearly supports the notion that ADAM9 is up-regulated on protein level in a larger proportion of renal cell carcinomas.

These findings are generally coherent with results from previous studies on ADAM9 in solid tumours which evidence the role of ADAM9 in tumourigenesis and tumour progression [22,25,51,52]. Functionally, blocking of ADAM9 with specific antibodies resulted in inhibited cell growth of gastric cancer cell lines [18]. Over-expression of ADAM9 in lung cancer cell lines resulted in enhanced invasiveness and was significantly associated with brain metastases [50]. In melanoma, ADAM9 is up-regulated in vivo at the invasion front [27]. In our study, the significant associations of ADAM9 with prognostically adverse conventional tumour parameters (positive nodal status, distant metastasis, residual tumour in the resection margins and higher tumour grade) are clearly in line with these findings.

Interestingly, ADAM9 was also found in most (13 of 17) papillary renal cell carcinomas. Although the number of cases with this subtype of RCC was small in our cohort, this information might be of adjunct diagnostic use for the assessment of renal cell carcinomas.

## Conclusion

In conclusion our results support the notion of ADAM9 to be associated with more aggressive tumours and unfavourable outcome. ADAM9 protein expression was significantly associated with shortened survival times but failed significance in a multivariate analysis. To further assess a possible independent prognostic role and the usefulness of ADAM9 for the sub classification of RCC, further validation using larger tumour cohorts is clearly warranted.

## Competing interests

The authors declare that they have no competing interests.

## Authors' contributions

FRF coordinated the study, performed immunohistological and statistical analyses and wrote the paper. KW supported the immunohistological evaluation and performed statistical analyses and wrote and revised essential parts of the paper. MoJ and AT performed the RT-PCR and contributed to the study design. IK contributed in the immunohistochemical analyses and array construction. ML, MaJ and MD provided samples and clinico-pathological data. KJ coordinated the study, provided samples and contributed to the study design. GK conceived and coordinated the study, performed immunohistological and statistical analyses, wrote and revised the paper.

## Pre-publication history

The pre-publication history for this paper can be accessed here:


